# The Global Session Metric Score (GSMs): A Modified Session-Specific Exertional Index

**DOI:** 10.3389/fspor.2021.692691

**Published:** 2021-07-27

**Authors:** Hanna M. Gardner, Andrew W. Scheck, John R. Cone, Nathaniel T. Berry, Laurie Wideman

**Affiliations:** ^1^Exercise Endocrinology Laboratory, Department of Kinesiology, School of Health and Human Sciences, University of North Carolina at Greensboro, Greensboro, NC, United States; ^2^Raleigh, NC, United States; ^3^Athletes Research Institute, Chapel Hill, NC, United States

**Keywords:** subjective monitoring, athlete monitoring, physiological load, perceived exertion, session-RPE, soccer, fitness periodization

## Abstract

Monitoring session training load to optimize the training stress that drives athlete adaptation and subsequent performance, is fundamental to periodization and programming. Analyzing the internal load experienced by the individual in response to the external load prescribed by coaching staff is crucial to avoid overtraining and optimize training adaptation. Subjective measures provide more information regarding individual training load, as heart rate measures alone do not account for collisions, eccentric muscle actions, muscle soreness, weather conditions, or accumulated training loads, which are paramount to the athlete experience. However, the current subjective metric for interpreting session training load (sRPE) is poorly shaped to the athlete's global response to the whole session, often showing poorer correlations to heart rate (HR) measures during intermittent or high-intensity activity. This study introduces a new metric, the Global Session Metric Score (GSMs), which creates a symmetrical relation between the verbal descriptor and numeric values, as well as more applicable session-specific verbal descriptors for the highest level of exertion. Twenty-four D1 male college soccer field players (age: 20.5 +/– 1.42) wore HR monitors and reported GSMs for all practices and games within an entire season. Linear regression with 10-fold cross validation was used to test the relation between GSMs with B-TRIMP and E-TRIMP, respectively. These models demonstrate good performance with consistency and reliability in the estimation of GSMs to predict both B-TRIMP (*R*^2^ = 0.75–0.77) and E-TRIMP (*R*^2^ = 0.76–0.78). The findings show promise for the GSMs index as a reliable means for measuring load in both training and matches during a high-intensity intermittent team sport. Future studies should directly compare GSMs to the existing sRPE scale within a controlled laboratory setting and across various other sports. GSMs provides coaches and clinicians a simple and cost-effective alternative to heart rate monitors, as well as a proficient measure of internal training load experienced by the individual.

## Introduction

Monitoring training load to optimize the training dose that drives athlete adaptation and subsequent performance, is fundamental to periodization and programming (Fry et al., 1991; Impellizzeri et al., 2004; Halson, 2014; Gabbett, 2016). Failure to balance training dose with recovery can have devastating effects on athlete performance and overall health and wellness. Simplistically, excessive training loads without adequate recovery, may lead to injury, illness, overtraining syndrome, and result in performance decrements (Fry et al., 1991; Smith, 2003; Coutts and Cormack, 2014; Halson, 2014; Saw et al., 2015), while insufficient training loads result in an athlete that is unprepared for competition and a commensurate increased risk of injury (Gabbett, 2016).

Training load can be conceptualized from two perspectives: (1) external load and (2) internal load. External load is the mechanical work completed by the athlete (Halson, 2014). This is usually the load of work prescribed by the coach, such as repetitions and sets or drills completed (Impellizzeri et al., 2005; Scott T. J. et al., 2013; Halson, 2014; Gabbett, 2016).

In intermittent team sport environments, external load is most frequently monitored through measurement devices such as global positioning systems (GPS), accelerometers, and highly advanced video analysis software (Halson, 2014). While useful for informing coaches about whether the athletes met the desired training prescription, it does not encapsulate the entire load and/or all of the factors that can impact training load—e.g., collisions, eccentric muscle actions, weather conditions, accumulated training loads—experienced by the athlete (Scott T. J. et al., 2013).

Internal load is the “relative physiological and psychological stress” imposed on the athlete through the external load performed (Halson, 2014, p. S141). This includes the cardiovascular, metabolic, neuromuscular, and psychophysical responses (Halson, 2014). In other words, the internal load—which is based on current fitness status—is how the individual athlete experiences the training, both physically and psychologically, which will ultimately determine their subsequent adaptation (Impellizzeri et al., 2005; Scott T. J. et al., 2013; Halson, 2014; Gabbett, 2016).

Optimizing individual athlete adaptation requires balancing the applied external load with the internal load experienced. This is influenced by athlete characteristics such as maturational age, training age, genetic background, and injury history (Gabbett, 2016), as well as the player's current fitness status, and training readiness inclusive of such elements as fatigue, mood, stress, and the dynamics of sleep (Halson, 2014; Jones et al., 2017). Individualization is especially relevant in team sports, where the primary focus is on team performance and thus training the team. The same training session results in varying external loads and divergent internal loads specific to each individual athlete. For this reason, monitoring internal training load of the individual athlete is crucial.

The positive linear relationship between heart rate (HR) and exercise intensity underlies the practical utility of monitoring physiological stress (i.e., internal load) (Karvonen and Vuorimaa, 1988) via HR. Based on this principle, heart rate monitoring technology has been a prominent means for measuring training intensity during exercise and thereafter calculating the training load of the session via “training impulse” (TRIMP)—a method first established by Banister and Calvert (1980). Derivations of TRIMP have continued, including the development of HR zones weighted on intensity, or based on lactate thresholds (Edwards, 1993; Lucía et al., 2000). The rapid fluctuations in intensity of work during intermittent exercise makes calculating training load from heart rate challenging—the lack of steady state exercise ultimately precipitates dissociation of the expected normative linear relationship between heart rate and work intensity. The nature of the relationship between heart rate and work during intermittent exercise is most simply described as resulting in “rhythmic fluctuations corresponding to changes in activity” (Drust et al., 2000, p. 891). Thus, while there are limitations to HR's application in measuring stress during intermittent sports it remains connected to the work performed at the primary avenue toward objectively quantifying internal training loads (McLaren et al., 2018).

The pursuit of a session measure was originated by Foster's adaptation of the Borg rating of perceived exertion (RPE) scale (Foster et al., 2001). Through his adaptation of Borg's 10-point RPE scale (Borg, 1982), Foster et al. (2001) created a subjective method for quantifying training load, one proven as a valuable tool for guiding exercise prescription (Foster et al., 2021). The quantification of a total training load value was achieved by multiplying the perceived difficulty of the session on the 10-point scale by the duration of the session in minutes to calculate session Rating of Perceived Exertion (sRPE). Thus, using Borg's CR10 RPE scale to replace heart rate, Foster created a TRIMP-like training load score “related to both HR and blood lactate markers of exercise intensity,” which also accounts for overall athlete well-being (Borg, 1982; Foster et al., 2001, p. 110).

The value of self-reported questionnaires lay not just in their utility and ease of use (Scott B. R. et al., 2013; Halson, 2014), but also in their ability to measure internal load that captures individual physiological responses to training load, and overall well-being (Saw et al., 2016). As physical training “imposes stress on an athlete, shifting their physical and psychological well-being along a continuum” with the potential to extend beyond acute fatigue toward overtraining (Saw et al., 2016, p. 1), subjective measures may provide additional nuanced information beyond training load. Since training apathy and decreased mood states are significant indicators of overtraining syndrome, establishing a snapshot of the athlete's psyche may be crucial to early detection of negative health outcomes and subsequent poor performance (Gabbett, 2016; Saw et al., 2016). Subjective measures have also proven to reflect acute and chronic training loads with superior sensitivity and consistency than objective measures (Saw et al., 2016).

The development of a more accessible, understandable, and accurate global metric of session load has been a constant in the pursuit of improving upon Foster's original work. This is highlighted by Foster's own statement that their work “has taken advantage of liberal modifications of Borg's original methods (Foster et al., 2021, p. 612).” These adaptations have included changes to the language, use of color coding, or providing pictured cartoons (Utter et al., 2004; Foster et al., 2021). The objective of the current research is to extend this process of iteration by specifically considering the athlete's internal response to a training session as a whole.

In this pursuit, the revised scale (global session metric) adapts the original Borg scale in two manners. The first adaptation leverages the 10-point scale's natural connectivity to 100% that is broadly accepted as the inherent scale for measuring human capacity for work in relation to the athlete's session experience. Specifically, the first objective is aligning the athlete's experience of the complete exhaustion of energy within the session requiring 100% of their total capacity. The result is a shift from Borg's identification of 10 as “maximal” to be newly phrased as “exhausting.” This shift is directly derived from the common English language definitions of maximal, defined as “an upper limit” versus exhausting which is defined as “to tire extremely or completely” or alternately to “consume entirely” (Merriam-Webster, 2021a,b). The foremost objective in this change is as Foster proposes, “to make the method more easily understandable and less dependent on standardized instruction (Foster et al., 2021, p. 612).” Further, the proposed semantics align to the following observation: “Defining [training load (TL)] as the physiological strain imposed on athletes, it may be assumed that exhaustion is the prevalent metric to determine the highest physiological strain, whatever the exercise, and that exhaustion should match the highest possible TL (Desgorces et al., 2020, p. 12).”

The second iteration to the scale is designed to reflect the player's session experience along a continuum as opposed to its original laboratory-based design intended to quantify exertion during graded exercise (Venhorst et al., 2018a). Specifically, as Borg designed his scale in a curvilinear manner (“Moderate” = 3, “Hard” = 5) to reflect lactate accumulation during acute graded exercise (Borg, 1982), by comparison the athlete's experience in a session is not experienced in a curvilinear manner. An athlete's experience in decision-driven sports is dynamic and unpredictable, characterized by repetitive bouts of varying intensities and durations, whereby physiological responses (i.e., heart rate and lactate levels) fluctuate. However, it has been shown that in intermittent sports such as soccer, development of fatigue is not directly linked to lactate accumulation or even glycogen concentration, acidity, or the breakdown of creatine phosphate (Westerblad et al., 2002). Additionally, other variables influence an athlete's internal load such as environmental factors, eccentric muscle actions, cognitive load, collisions/impacts, cumulate over the session and are likely not experienced in a curvilinear manner in the training session.

For these reasons, the cumulation of load, viewed as the sum of these demands, upon session cessation by the athlete (representing the training load) is more logically represented linearly in relation to maximal physiological capacity (i.e., exhaustion). To better orient the player to capacity existing along a spectrum, the verbal descriptors were aligned accordingly with “5” as the mid-point in the scale changing to “moderate.” A further adjustment was to balance the scale along 10-points to more completely align with the player's experience of the session. The selection to leave certain numerical values without descriptors was to limit challenges to the athlete's interpretation. For instance, with 7 and 8 described as “hard” and “very hard,” respectively, the descriptor of 9 is intentionally blank to avoid challenges of nomenclature and subsequent interpretation of such phrasing as “very, very hard” or “extremely hard.”

To our knowledge this iteration of Borg's original scale is the first subjective metric to systematically address the player's session-specific experience. Therefore, the aim of this study was to investigate the validity and reliability of the new Global Session Metric Score (GSMs) in quantifying session specific exertion in male college soccer players relative to objective HR-based metrics of internal training load. We hypothesized that the GSMs will be highly correlated with HR measures [e.g., Edward's TRIMP (E-TRIMP) and Banister's-TRIMP (B-TRIMP)] for training, games.

## Methods

### Subjects

The final dataset used in these analyses consisted of 24 field players (age = 20.5 ± 1.42 years, ht = 188.8 ± 4.77 cm, wt = 73.2 ± 7.29 kg) with a total of *N* = 1,274 observations. Out of the 25 field players' available data, one player was excluded from analysis due to missing data (45 combined training and game days). Due to differences in the demands of training and match play between field players and goalkeepers, all goalkeeper data was excluded from this study. Each player in the study had multiple years of experience playing soccer at the highest youth levels within their respective states and countries. The players' experience in NCAA Division I soccer was 1.92 years (SD = 1.06 years). A total of 10 players had no previous experience, seven had 1 year of experience, two had 2 years, and three had 3-year previous experience. Prior to the start of the season, and this study, all returning players were re-familiarized and incoming players familiarized to wearing HR monitors and using GSM. The majority of participants were familiar with wearing HR monitors and reporting GSMs during their previous years' experience.

### Data Collection

The study spanned the length of the season, 13 weeks, with data collected at every field-based session and match, totaling 71 days.

To maximize comparisons to previously published data, TRIMP was calculated using the equations from both Banister (B-TRIMP) (Banister, 1991) and Edwards (E-TRIMP) (Edwards, 1993). To properly calculate both TRIMP equations, the maximal HR (HR_max_) and resting HR (HR_rest_) were collected for every athlete. The Yo-Yo Intermittent Recovery Test Level 1 (YYIRL1) was used to determine each participant's HR_max_ (Krustrup et al., 2003), while the resting HR (HR_rest_) was assessed during a 10-min period with the athletes in a supine position in a dark room prior to eating breakfast.

Heart rate data was collected via live telemetry (Firstbeat Sports, Finland) for every athlete from each field-based session throughout the season. The data was analyzed as a %HR_max_, B-TRIMP, and E-TRIMP. Each TRIMP equation produces a single value for the training load of a session, expressed in arbitrary units, which was used to compare to the GSMs value for the session.

The Global Session Metric (GSM) value of the training session was reported after each field-based session and match, via Fit for 90 (FF90; a web-based athlete monitoring application). Each player had a personal login for the application where they were able to report their GSM and specify what kind of session was performed (i.e., “Team Training,” “Match”). At the beginning of the season, FF90 staff downloaded the app onto each player's smartphone. All phones were left inside the locker room while players were out on the field. This ensured a 15- to 30-min window post-training before players reported their GSM within the app. This method maximized privacy for reporting the GSM, avoiding the bias and pressure that accompanies verbally reporting scores to a coaching staff member or individuals writing down scores on paper passed around to everyone.

Using the GSM scale, athletes rated the intensity of each session. The session duration, defined as the start of the warm-up through the end of the training session (with cool-down excluded), was carefully monitored and recorded by athletic training staff, and uploaded into the app after each session. For matches, coaching staff submitted the total number of minutes played in the match for each player for their training duration, pre-match warm-ups were excluded from the match duration (Gil-Rey et al., 2015). The submitted GSM number reported by the athlete along with the duration of training/game playing time was used by the app to calculate the GSMs. All data was compiled in the FF90 application into a single dashboard for use by the coaching staff during the season. At the end of the season, FF90 staff exported all de-identified data (ID numbers only) into a CSV file for subsequent analysis by the research team (secondary data analysis; IRB #14-0439).

### The Global Session Metric Score

**Figure 1:**
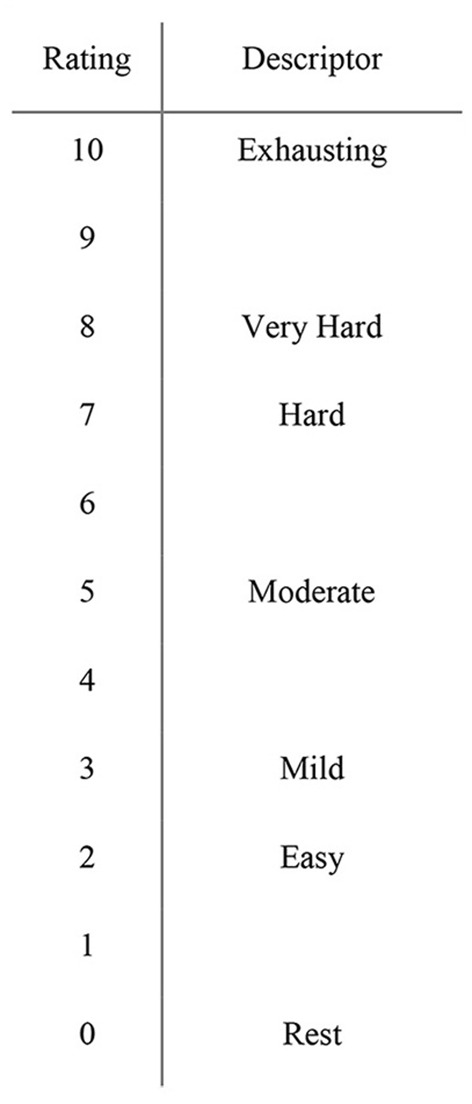
The new Global Session Metric (GSM) scale.

The modified RPE scale (GSM) establishes a symmetrical relation between the verbal descriptors and numeric values [compared against Borg (1982) CR10 in Figure 1], with “3” classified as “Mild” and “5” as “Moderate.” In place of the previous indicator of the highest level of exertion (“10”) as “Maximal,” the verbal descriptor “Exhausting” is used.

The final GSMs value was calculated by multiplying the subjective rating, GSM, by the total number of minutes trained/played.

GSMs is calculated as,

$$\begin{array}{l} GSMs=\;subjectiveRating\;*\;minutesPlayed \end{array}$$

### Statistical Analysis

Reliability of GSMs was examined using repeated measures correlations which account for the non-independence of observations within individuals. Similar to simple linear correlation, repeated measures correlations measure the strength of the relation between two variables, but accounts for the inter-individual variability. To examine reliability in GSMs in training and game play, we examined the correlations between GSMs~B-TRIMP and GSMs~E-TRIMP during training, game play, and in a combined/full dataset.

Data were normalized and scaled by taking the square root and dividing by a fixed scalar. The transformations used for GSMs, B-TRIMP, and E-TRIMP are,

$$\begin{array}{l} \;GSMs_{scaled}=\;\frac{\sqrt{GSMs}}{4}\\ BTRIMP_{scaled}=\;\frac{\sqrt{BTRIMP}}{2.2}\\ ETRIMP_{scaled}=\;\frac{\sqrt{ETRIMP}}{3} \end{array}$$

These transformations are implemented to normalize the data and place each of these variables on similar scales.

Separate models to predict B-TRIMP and E-TRIMP, were developed using linear regression with 10-fold cross validation. To avoid issues of dependence between samples, observations of GSMs~B-TRIMP and GSMs~E-TRIMP were randomized prior to analysis; the same randomization scheme was used for both GSMs~B-TRIMP and GSMs~E-TRIMP. Model performance across each of the 10-folds was assessed using the coefficient of determination (*R*^2^) and root mean square of error (RMSE). The RMSE for the transformed variables provides a method of comparing performance between models while the RMSE of the estimates in the original units are provided for orientation and alignment for those more familiar with the B-TRIMP and E-TRIMP values. A final model was created by averaging the parameter estimates from each of the 10-folds.

## Results

Repeated measures correlations show a similar significant positive relation between GSMs and B-TRIMP, and GSMs and E-TRIMP for the full, training only, and game only datasets (Table 1). These findings indicate good reliability in GSMs to predict B-TRIMP and E-TRIMP in both training and game play.

**Table 1:** Repeated measures correlations between GSMs, B-TRIMP, and E-TRIMP.

**Relationship**	**Coefficient median**	**df**	**95% Confidence interval**	***p*** **-Value**
GSMs~B-TRIMP (full dataset)	0.88	1132	0.87–0.90	<0.001
GSMs~B-TRIMP (training only)	0.88	889	0.87–0.90	<0.001
GSMs~B-TRIMP (game only)	0.85	219	0.81–0.88	<0.001
GSMs~E-TRIMP (full dataset)	0.89	1132	0.88–0.90	<0.001
GSMs~E-TRIMP (training only)	0.88	889	0.86–0.89	<0.001
GSMs~E-TRIMP (game only)	0.86	219	0.82–0.89	<0.001

*The range of coefficients and p-values for the game only dataset is influenced by the low number of observations for some individuals. df, degrees of freedom*.

**Figure 2:**
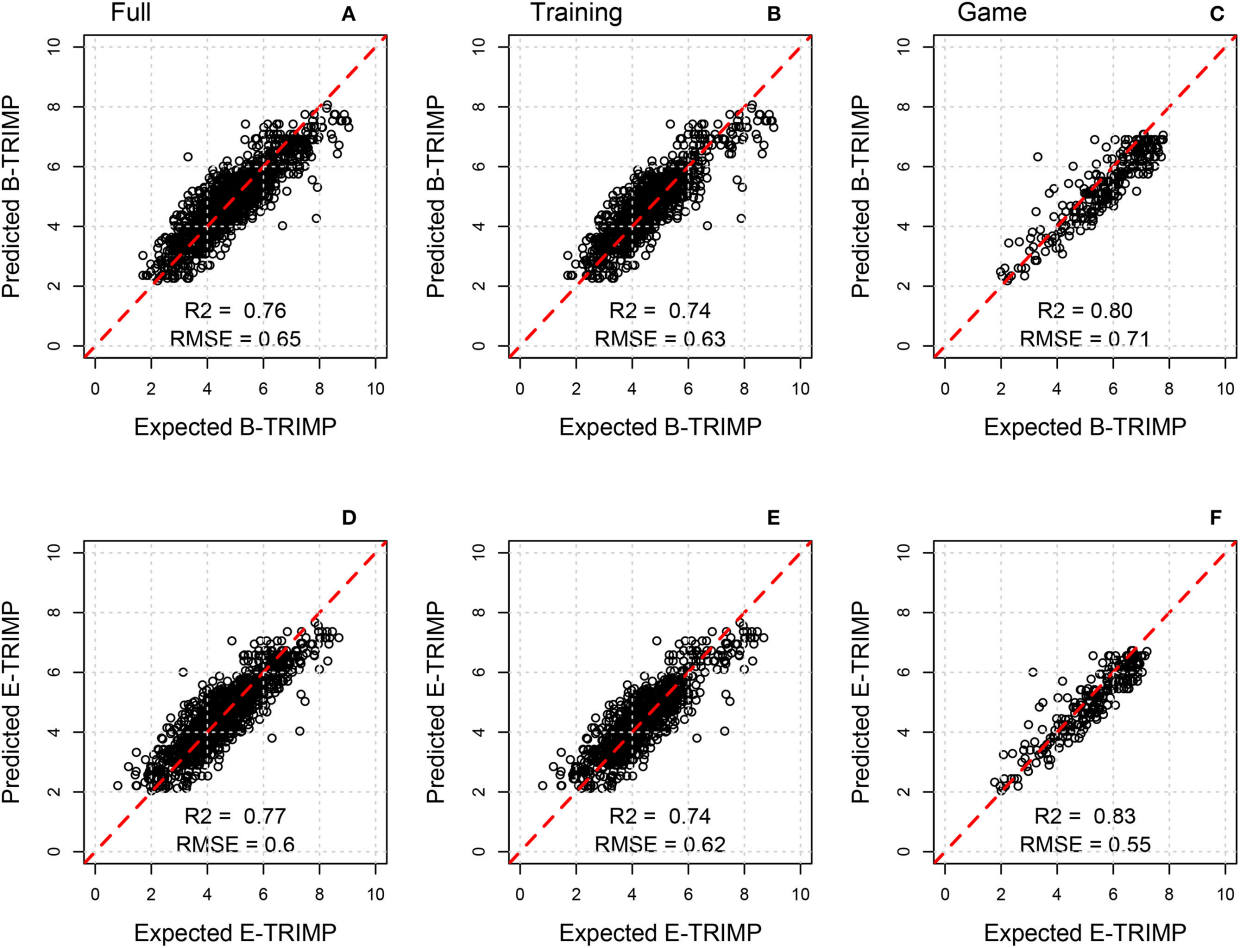
Predicted vs. Expected values for B-TRIMP and E-TRIMP using the final prediction models. Data are presented as a full dataset **(A,D)**, a training dataset **(B,E)**, and game dataset **(C,F)**. Coefficient of determination (*R*^2^), and root mean square error (RMSE).

The 10-fold cross validation analysis was used to demonstrate the predictive strength of GSMs. The results (Table 2) show consistency and reliability in the estimation of GSMs to predict both B-TRIMP and E-TRIMP (Figure 2). The model fit of B-TRIMP (*R*^2^ = 0.75–0.77) and E-TRIMP (*R*^2^ = 0.76–0.78) demonstrates good model performance and establishes the validity of GSMs to predict B-TRIMP and E-TRIMP. RMSE for B-TRIMP (RMSE = 0.59–0.70 scaled A.U., 28.6–36.6 A.U.) and E-TRIMP (RMSE = 0.55–0.66 scaled A.U.; 46.1–58.7 A.U.) indicates low error in prediction.

**Table 2:** Model performance, assessed via the coefficient of determination, root mean square error (RMSE) of scaled units, and RMSE of original units for the prediction of GSMs~B-TRIMP and GSMs~E-TRIMP using linear regression with 10-fold cross validation.

		**1**	**2**	**3**	**4**	**5**	**6**	**7**	**8**	**9**	**10**
B-TRIMP	*R* ^2^	0.76	0.76	0.75	0.76	0.76	0.76	0.76	0.76	0.77	0.76
	RMSE (scaled units)	0.67	0.61	0.63	0.67	0.67	0.61	0.67	0.67	0.7	0.59
	RMSE (B-TRIMP units)	32.0	30.3	35.2	36.6	35.5	28.6	36.3	32.6	36.3	29.0
E-TRIMP	*R* ^2^	0.77	0.77	0.76	0.77	0.77	0.77	0.77	0.77	0.78	0.77
	RMSE (scaled units)	0.65	0.57	0.58	0.63	0.6	0.59	0.58	0.63	0.66	0.55
	RMSE (E-TRIMP units)	53.8	46.4	54.1	58.7	52.8	47.8	53.2	54.6	58.3	46.1

A final model was determined by averaging the estimates from each of the 10-fold cross validation iterations. The final models for B-TRIMP and E-TRIMP are presented as:

$$\begin{array}{l} {B\;TRIMP}_{est}\;=\;0.72657\;*\;GSMs\text{\_}scaled\;+\;1.45392\\ {E\;TRIMP}_{est}\;=\;0.69534\;*\;GSMs\text{\_}scaled\;+\;1.34204 \end{array}$$

The GSMs proves to be reliable and valid within a high-intensity intermittent environment such as soccer, showing strong significant positive relations between GSMs and B-TRIMP and E-TRIMP (Table 1) as well as high prediction accuracy (Table 2) within training and game play.

## Discussion

The results of the current study validate the use of the GSMs index as an efficient and effective measure of overall (i.e., global) session training load. This is foremost highlighted via the predictive model's ability to accurately reflect overall exertion across games, training, and combined environments. Further, given the strong correlations to TRIMP, the GSMs is proven to be a valid means for measuring load in both training and matches during a high-intensity intermittent team sport, such as men's DI soccer. GSMs provides coaches and clinicians a simple, easy-to-interpret method of estimating training load with the use of a subjective questionnaire converted into the GSM score. We lay out below the methods by which GSMs may be applied as a framework for coaches to periodize their season and adapt training load given the feedback loop created by the GSMs reporting to best serve the individual athlete to optimize performance adaptation and reduce injury or overtraining.

### GSMs and HR Metrics

The results from our analyses show the subjective scaling method (the GSMs) of the session-specific experience to reliably align with the objective heart rate measures (e.g., B-TRIMP and E-TRIMP). Thus, the high correlation between GSMs and TRIMP proves the strength of the subjective measure as a surrogate, alternative, or supplementary metric to the objective measurement of loads via heart rate. For teams that cannot afford expensive heart rate monitoring equipment, GSMs is a cost-effective, easily implemented, and accurate measurement tool for tracking training load. For teams that can afford objective load measurement tools the accessibility of the GSMs facilitates the use of a highly accurate tool that is more accessible for the coach's and practitioner's development and implementation of target training loads.

It is important to consider the origin of Foster's sRPE utilizing the Borg scale's original design toward quantifying effort during acute bouts of exercise and transferred to the context of the session (Venhorst et al., 2018b). Specifically, using a scale that was originally created to represent the curvilinear nature of lactate accumulation within a single acute bout of increasingly intense exercise to a longer-term session will be accompanied by a decrease in accuracy. The development of the GSM was driven by the desire to establish a revised scale with the specific purpose to encapsulate the global experience felt by the athlete due to a training session. Establishing a symmetrical relation between the verbal descriptors and the numeric values in the GSM scale is a more accurate reflection of a player's experience across an entire session. The athlete's perception of demand is session dependent, and specific to the progression and ebb and flow or workload(s) throughout the session. Specifically, the type of work performed, and foremost its intensity and volume, when it occurs in the session, combined with the potential dynamic unpredictability of sport, all interact to influence the player's session experience. The constant across sessions is the sum of the player's session experience quantifiable through their perception of the cumulative demand required in the session. Changing the verbal descriptor used by Borg to describe peak exercise intensity—“Maximal”—to better represent the session experience, “Exhaustive,” as discussed, was implemented to better connect with the player's experience of the session. These qualities of the GSMs likely contribute to the high correlations with TRIMP documented in the current study.

Despite the utility of the GSMs shown here, further research and efforts to improve the accuracy of the global measurement via subjective scales is needed. The remaining discrepancy between GSMs and TRIMP should be viewed as a challenge to the scientific community to achieve change where room for improvement remains. Future research should more comprehensively consider factors influencing the player's experience. For instance, research examining GSMs accuracy in relation to changes in training status, or the inclusion of analyses of physical environmental factors, such as ambient temperature, that are known to influence both HR and perception of exertion (Galloway and Maughan, 1997) will facilitate greater understanding of both player perception and improve the development of subjective monitoring systems. Further, direct comparison of GMSs with sRPE and other subjective and objective measures of training loads is essential. While the comparison to sRPE would have been ideal in the current study, the reality of the performance environment, or any research study for that matter, is the delicate balance between ideal study design and acceptable participant burden—delivery of an additional training metric was not possible in the current study.

Future analysis should consider interconnected factors accompanying sport-specific to non-specific training that may influence objective and subjective measures of training load. For instance, recent analysis of high-intensity interval training via running vs. small-sided games demonstrated athlete's report greater enjoyment during small-sided games (Selmi et al., 2020). This leads to the potential that while similar physiological stress may occur between two sessions, the player's perception of the stress may vary according to the dynamics of the player experience. Further, as research examining the combined effects of exercise with cognitive demands has demonstrated an interaction effect on fatigue (Chatain et al., 2019), it is important to consider the role of the combined physiological stress within a session with the cognitive stress accompanying player decision-making that is both connected to the sport, but also impacted by the coach. Considering factors beyond the session, such as the entire week of activities, research has shown that player perception of training load was connected to the results the player's achieved on the prior weekend, where a negative (i.e., loss) versus positive (i.e., win) resulted in higher and lower perceived training loads, respectively (Brito et al., 2016). This latter study highlights the potential that factors external to the player's training experience itself may influence the player's perception of the session's difficulty.

### GSMs as a Metric of Internal Training Load

While the current study demonstrates the GSMs' association with HR-derived TRIMP, we propose that GSMs (as a subjective measure) may provide a more accurate global metric of internal training load experienced by the athlete than objective metrics alone. Further, GSMs provides a platform for the coach and practitioner that is highly practical for the (1) production of a periodization plan and (2) monitoring of the periodization's implementation via athlete monitoring and coach's adaptations.

Using subjective metrics alongside objective metrics for monitoring athlete load provides greater insight to the athlete's global response to a training session. While HR is well-documented to have variable responses to heat stress and cold conditions during exercise (Brooks et al., 2005) as well as accumulated training loads accompanying changes in training status (Zavorsky, 2000), other factors such as collisions, types of muscle actions, illness, injury, mood, or cognitive load may all impact an individual's internal load without detection by objective metrics. Furthermore, subjective metrics like GSMs encompass the cognitive-emotional experience of the player in the session that is inherent to team-based sport, driven dually by player-player interactions and coach-player interactions. Finally, psychological stressors associated with strenuous physical exertion, performance anxiety, and outside influencing pressures are known to increase an athlete's RPE (Morgan, 1973; Brito et al., 2016). While these elements have been quantified via measurements analyzing the dynamics of physiological stress at rest (i.e., heart rate variability and blood pressure) they have yet to be examined during exercise. Given the dynamics of heart rate control during exercise, the changes observed at rest may be less detectable or even concealed during exercise, although no less influential. These gaps in both HR as an intrinsic measure, and the variable accuracy of GPS in relation to the type of work performed (Crang et al., 2020), an accurate measure of subjective training load is a crucial element of effective player management (Halson, 2014).

The elevated accuracy of internal load via the GSMs, used instead of, or alongside, the objective benchmark of TRIMP is a positive step forward in the establishment of progressively valuable tools where optimizing athlete performance and mitigation of risk for injury and/or overtraining is a fundamental objective of monitoring (Saw et al., 2016).

Future studies should investigate whether the variance between GSMs and TRIMP can be attributed to the quantity of accelerations and decelerations performed, weather conditions, potential psychological stressors (i.e., final exams, bigger stages of competition, etc.), or the accumulation of load over multiple training days.

### Practical Application of GSMs

Implementing GSMs into a team's methods for measuring training load is simple and practical at all ages and skill levels, and across various sport environments. However, to ensure reliability, correct procedures must be followed. The accuracy of any subjective measurement is predicated on the culture created by the coach, and the subsequent use of the information in managing the player. It is paramount that the coach and practitioner consider their influence and role in collecting accurate subjective monitoring data.

First, conversations with coaching staff can be framed in a simple, easy to understand manner around GSMs, providing them with a target for the desired athlete load and subsequent training response. For example, a coach may be asked to design a training session with a difficulty of a “7 or 8” (based on the GSMs 0–10 index) which will last 70 min. This can be conceptualized and implemented more easily by a coach compared to the more abstract idea of planning a training session that induces the proper HR-derived TRIMP score. Second, following implementation of a training session, feedback from the players regarding the coach's effectiveness in achieving the target GSMs is immediate, and in essence answers the coach's question “did I hit the mark” in a direct and highly accessible manner. Subsequently, the feedback loop that is created informs the coach's future decision-making and improves the coach's own understanding and methods. In this manner, the simple format and structure of the GSMs provides the ideal platform and methodology to periodization planning.

Accuracy of any subjective scale is dependent on players being properly accustomed to the scale and informed of the scale's purpose. In terms of best practices for the session scale's implementation, since the last activity of the training session has the tendency for largest influence, it is important that the GSMs is administered after sufficient cool-down, 15–30 min after the completion of practice (Hornsby et al., 2013; Uchida et al., 2014; Fanchini et al., 2015; Scantlebury et al., 2018). Ideally, the reporting would occur in a private setting (such as a web-based application), without influence or pressure by other teammates or coaching staff (Roos et al., 2018).

With the objective of optimizing each individual athlete's performance adaptations, and the increased chance to avoid injury, illness, and overtraining (Fry et al., 1991; Saw et al., 2016), the accuracy of monitoring via GSMs has the capacity to aid in the practical implementation of accurate training. Over the course of a season the GSMs allows coaching staff to more readily (1) align training loads to the periodization plan, (2) make day-to-day adaptations to prescribed training loads and/or athlete playing time based on team or individual scores, and (3) monitor individual athletes' global training response.

The accessibility and accuracy of daily GSMs of players' internal loads provides coaches with valuable information enabling decisions regarding the demands of their sessions, and the need to alter future training sessions based on the prior scores. For example, if an individual athlete reported a GSMs signifying a large internal load to the previous day's training session, the coach may decide to alter the individual's role(s) in the next day's training to effectively “lighten the load” with the intention of reducing the risk of overtraining, or alternately, limit that individual's participation time in practice.

### Limitations and Strengths of the Current Study

Despite the large number of observations through an entire season, the data were collected on a single D1 men's soccer team and future studies should include a larger sample, including women's teams and teams of other levels of competition (i.e., recreational, pro) as well as other sports.

The largest limitation of this study is that sRPE was not collected, so there is an inability to directly compare GSMs and sRPE for each training session or match.

This study was also completed within a variable performance environment (i.e., men's soccer practices and games with accumulating training loads across a season and located outdoors with varying environmental factors). Future laboratory-based studies should be done that control for session demands which would ideally compare GSMs to sRPE, and both of these to TRIMP.

### Conclusion

The GSMs not only allows quantification and tracking of training load, but also provides a tool to establish a methodology capable of fine-tuning and easily adjusting the athlete's programming within the periodization plan. The foremost objective of the GSMs is to better encapsulate the athlete's experience over an entire training session, with a linear relation between the verbal descriptors and numeric values. The current data suggest that the modified RPE scale used in the GSMs allows athletes to accurately report scores of global internal training load experienced during a training session, thus accurately quantifying internal training session load. In conclusion, our data suggests that the GSMs is accurate in measuring internal training load with high correlations to B- and E-TRIMP. This method is a simple, easy and cheap global metric for evaluating an athlete's response to training. The GSMs index can be used to align an athlete's training with a periodization plan, signify the coach to adapt training session load, or ring alarm bells to an athlete's overall well-being in response to training or progression toward overtraining syndrome. Overall, the GSMs provides a practical effective platform for establishing periodization methods and optimizes training loads that maximizes athlete development, performance, and injury resistance.

## Data Availability Statement

The raw data supporting the conclusions of this article will be made available by the authors, without undue reservation.

## Ethics Statement

The studies involving human participants were reviewed and approved by Ethics Committee of the University of North Carolina at Greensboro. The patients/participants provided their written informed consent to participate in this study.

## Author Contributions

JC: creator of the novel training load scale, conceptualization, and methodology. AS: data collection, data cleaning, and subject demographics. NB: statistical analysis and writing of the results section. HG: lead author of the manuscript. LW, JC, NB, and AS: review and editing. All authors contributed to the article and approved the submitted version.

## Conflict of Interest

The authors declare that the research was conducted in the absence of any commercial or financial relationships that could be construed as a potential conflict of interest.

## Publisher's Note

All claims expressed in this article are solely those of the authors and do not necessarily represent those of their affiliated organizations, or those of the publisher, the editors and the reviewers. Any product that may be evaluated in this article, or claim that may be made by its manufacturer, is not guaranteed or endorsed by the publisher.
